# A Pilot Feasibility Study Exploring the Preliminary Effectiveness of an AI‐Driven Virtual Human Intervention for General Practitioner Obesity Education and Communication‐Skills Training

**DOI:** 10.1002/osp4.70083

**Published:** 2025-07-08

**Authors:** Leona Ryan, Sean Coleman, Triinu Zimmermann, Rory Coyne, Elizabeth Broadbent, Anne Browne, Grainne O'Donoghue, Fiona Quigley, Hemendra Worlikar, Cornelia Connolly, Michael Crotty, Susie Birney, Owen Conlan, Jane C. Walsh, Derek O'Keeffe

**Affiliations:** ^1^ School of Psychology University of Galway Galway Ireland; ^2^ Health Innovation via Engineering Laboratory University of Galway Galway Ireland; ^3^ Department of Psychological Medicine The University of Auckland Auckland New Zealand; ^4^ Medicine College of Medicine Nursing and Health Sciences University of Galway Galway Ireland; ^5^ School of Public Health Physiotherapy and Sports Science University College Dublin Dublin Ireland; ^6^ School of Communication and Media Ulster University Belfast UK; ^7^ School of Education College of Arts, Social Sciences, & Celtic Studies University of Galway Galway Ireland; ^8^ Irish College for General Practitioners (ICGP) Dublin Ireland; ^9^ Irish Coalition for People Living with Obesity (ICPO) Dublin Ireland; ^10^ School of Computer Science and Statistics Trinity College Dublin Dublin Ireland

**Keywords:** Artificial intelligence, medical education, obesity, person‐centred care

## Abstract

**Background:**

Rising global obesity rates demand effective weight management strategies from general practitioners (GPs). However, time constraints, training gaps, and low confidence often impede GPs' ability to conduct weight‐based conversations. This pilot study assessed the feasibility and preliminary effectiveness of an AI‐driven Virtual Human (VH) obesity education and communication‐skills training tool, specifically designed to address these challenges and enhance obesity education and communication‐skills among GPs.

**Methods:**

A pilot feasibility study with a pre‐post survey design evaluated the impact of the VH tool on knowledge, self‐efficacy, empathy toward patients with obesity, and confidence in clinical consultations. Participant perceptions, trust, and intention to use the VH tool were explored. Paired‐sample *t*‐tests were conducted to evaluate within‐group mean differences. Descriptive statistics were used to evaluate feasibility and acceptability.

**Results:**

A total of 22 GPs were recruited. Despite some attrition, significant improvements were observed in knowledge (*p* = 0.006), self‐efficacy (*p* = 0.001), and combined empathy and confidence scores (*p* = 0.002). Alongside these improvements, participants demonstrated positive perceptions of the tool, high trust in the VH, and a strong intention to implement the learned strategies.

**Conclusions:**

This pilot study demonstrates the potential of an AI‐driven VH tool to enhance GP obesity education and communication skills. The observed improvements in key outcomes support the potential of VH technology in medical education on obesity. To further establish the efficacy and explore the broader applicability, future research should focus on larger, controlled trials across various provider groups. Overall, these preliminary observations highlight a promising avenue for enhancing the skills of a wider range of providers in the obesity treatment space.

## 1 Introduction

Obesity presents a significant and complex challenge within general practice settings, requiring general practitioners (GPs) to take a leading role in the identification and management of the disease, and counseling patients on personalized treatment options [1, 2]. Clinical obesity practice guidelines [2, 3] recommended a person‐centered approach to obesity treatment, initiating with the 5A's framework [4] to facilitate the collaborative exploration of individualized treatment options. However, the implementation of this recommendation continued to face challenges, as GPs reportedly circumvented the initiation of weight‐related conversations with their patients [5, 6] due to time constraints, limited access to educational resources [7, 8], and a lack of confidence and skill in addressing weight‐related conversations [9]. Specifically, GPs reported feeling ill‐equipped to navigate sensitive discussions around weight management [6], manage patients' emotional responses, or effectively counsel beyond basic lifestyle advice due to insufficient practical training [5, 6]. Furthermore, existing educational resources are often perceived as infrequent, didactic [7, 8], or difficult to integrate into busy clinical schedules [6]. This often meant that comprehensive, empathetic, and patient‐centered weight management discussions were either brief or entirely absent from consultations, thereby hindering effective care [6, 9].

Paradoxically, patients with obesity expressed a willingness to engage in weight‐based discussions with their GPs [9, 10]; however, the manner in which these conversations were conducted significantly influenced the perception of weight stigma [5, 9]. Consequently, patients with obesity often experience weight stigma, which subsequently undermined the patient‐provider relationship [10] and negatively impact health outcomes [11, 12]. Digital interventions aimed at bridging this communication gap have demonstrated limited success. For example, Welzel et al. [13] highlighted the potential of online 5A's tutorials, but revealed critical shortcomings, including a lack of robust theoretical foundation, hindering the evaluation of its acceptance, feasibility, and usability [14]. Additionally, the absence of a theoretical framework limited the study's ability to effectively measure and analyze the intervention's impact. Therefore, there was a clear and pressing need for theoretically sound, practically feasible interventions that empowered GPs to deliver effective, person‐centered care for obesity.

Artificial intelligence (AI) offers a promising solution for addressing this training gap. AI‐driven tools provide accessible, flexible, and personalized learning experiences tailored to the specific needs of GPs [15, 16]. These tools could overcome the limitations of traditional training methods by offering on‐demand access and consistent content delivery. Specifically, AI‐driven VHs present a sophisticated medium to facilitate structured learning experiences, accelerating the development of expertise in delivering sensitive weight‐related conversations. Virtual humans can facilitate skills‐based practice through simulated patient interactions, allowing GPs to develop and refine their communication strategies in a safe and controlled environment [17, 18].

Virtual human platforms, defined as AI‐driven technologies that create interactive digital avatars capable of simulating human‐like behavior and communication, offer unique affordances as medical education tools, providing unlimited opportunities to practice clinical skills and receive feedback in a realistic and safe environment. They standardized clinical experiences and promoted mastery by exposing users to a diverse range of clinical presentations, reinforcing understanding of underlying principles, and enabling informed decision‐making [16, 18, 19]. Furthermore, recent research indicated that VHs, leveraged as behavioral models in digital learning settings, enhanced learners' self‐efficacy beliefs in their ability to perform tasks [20]. Therefore, AI‐driven VHs hold significant promise in addressing the challenges GPs face in delivering person‐centered obesity care. By exposing GPs to a variety of patient scenarios, VHs could enhance self‐efficacy, empathy, understanding of obesity‐related principles, and enable the development of effective weight‐related communication strategies.

This pilot feasibility study explored the feasibility and preliminary effectiveness of AI‐driven obesity education and communication skills training modules with VH delivery for GPs. The modules were developed following the intervention mapping protocol [21] and incorporated behavioral science frameworks, including the Behavior Change Wheel [22] and the Theoretical Domains Framework [23], selected for their established utility in designing and evaluating behavior change interventions in healthcare settings. To further enhance effectiveness, behavior change techniques (BCTs) [24] were integrated into the VH design features. A detailed explanation of how these frameworks were applied in the intervention design is provided in the methods section. Although AI has shown promise in other areas of medical training [15, 18], its application to obesity education and weight‐based communication training for GPs has been underexplored. Therefore, to address this gap, the objectives of this pilot feasibility study we explored: (1) the feasibility of an AI‐driven obesity education and training tool with VH delivery for general practitioners; (2) the preliminary effectiveness of its use on knowledge acquisition, self‐efficacy, empathy toward patients with obesity, confidence in clinical interactions, and intention to implement learning in future interpersonal interactions with patients with obesity; and (3) the potential for future use of an AI tool for training and education purposes in this context. Given the pilot nature of this feasibility study, formal hypotheses were not developed. Instead, we aimed to assess preliminary evidence of effectiveness in the outcome measures to inform the feasibility of a future randomized control trial, including sample size calculations.

## 2 Methods

### 2.1 Design

A pre‐post survey design was used to evaluate the feasibility and preliminary evidence of effectiveness of an AI‐driven educational tool designed to support obesity education and communication skills training among general practitioners. The study is reported following the Consolidated Standards of Reporting Trials (CONSORT) statement: extension to pilot and feasibility trials [25]. The pilot feasibility study was conducted online via Qualtrics from February 2024 to July 2024.

### 2.2 Intervention Development

The VH delivered intervention was developed using the Intervention Mapping protocol [21]. The Intervention Mapping protocol offers a systematic, six‐step framework for building effective interventions grounded in theory and evidence [26]. The approach commenced with a needs assessment, incorporating perspectives from both patients and practitioners to define the intervention's objectives and target behaviors. The Behavior Change Wheel (BCW) [22], the Theoretical Domains Framework (TDF) [23], and the Behavior Change Taxonomy [24] were utilized to identify the theoretical constructs to include in the intervention prototype to address the target behaviors. A multidisciplinary planning group, comprising researchers, clinicians, behavioral scientists, engineers, a health communication expert, and individuals with lived experience of obesity, guided the translation of the theoretical concepts into practical components to include in the intervention. A detailed overview of the Intervention Mapping process and subsequent intervention development is reported elsewhere [27].

### 2.3 Intervention Content

The intervention featured an AI‐driven VH presented as a GP delivering theoretically‐informed [27] audiovisual, interactive educational and skills‐training modules. The two modules of the VH intervention were self‐paced. While the total completion time for both modules ranged from 30 min to a maximum of 45 min in this pilot, individual engagement and learning speed influenced the duration. The approximate duration for each module was 20 min. The first module consisted of an evidence‐based module that outlined the etiology of obesity and weight stigma [2, 3]; the second module delivered an interactive skills‐training module on the 5 As (Ask, Assess, Advise, Agree, Assist) five‐step framework for weight management communication [4]. The intervention was hosted on a controlled platform, for precise testing and adjustments. Module content was delivered to users via a pre‐defined script by the virtual GP. Each step of the 5 As framework was outlined within the script. Following each step, users completed a short quiz. The virtual GP responses to user choices were pre‐programmed to adhere strictly to the script, ensuring consistency during testing. The content was reviewed and approved by members of the planning group.

### 2.4 Technical Design

The intervention was delivered using a VH developed with AI software from Soul Machines (https://www.soulmachines.com/creator‐tools/). This VH was configured for human interaction simulation in education and communication‐skills training. The intervention design integrated a rule‐based chatbot for content delivery with an AI‐driven VH avatar. The components and functions of the VH are detailed in Table 1. The VH's verbal dialog was scripted by the research team. During interactions, VH verbal responses were selected from this script based on trigger word detection in user responses. IF, AND, OR, and NOT statements defined response selection. If no trigger words were detected, the VH prompted rephrasing; if not understood after a second attempt, the VH would apologize and move on, offering on‐screen prompts at a later point to recap using frequently asked questions. This system provided controlled consistency for the pilot trial.

**TABLE 1:** AI features and application in the intervention.

AI feature	Description	How AI is used
Pre‐defined script modelling communication behaviours	The virtual human followed a scripted dialog delivering evidence‐based education and communication techniques for discussing obesity with patients.	Script authored by experts was used as input; the AI drove delivery and adapted responses dynamically based on user interactions.
Text‐to‐speech synthesis	The AI converted the script into natural, human‐like speech in real time.	AI‐generated voice simulated tone, pace, and rhythm of empathetic spoken language to enhance realism.
Lip synchronisation	The virtual human’s mouth movements were synced with the spoken words.	AI mapped phonemes to visual mouth shapes, providing realistic lip movement and improving immersion.
Emotionally responsive facial expressions	Facial expressions (e.g., smiling, concern) were triggered to match the script’s emotional content.	AI‐driven animation aligned facial expressions with the underlying sentiment of the script or user’s selected responses.
Voice tone and emotion congruence	Voice tone changed to reflect appropriate emotions (e.g., warmth, concern, encouragement) during interactions.	AI adjusted vocal characteristics (pitch, intonation) to match intended emotional tone, ensuring consistency with visual cues.
User‐interaction response adaptation	The virtual human reacted to user‐selected answers (e.g., quiz or dialogue choices) in real time.	AI enabled branching responses or adaptive reactions based on user input, maintaining conversational flow and emotional coherence.

**FIGURE 1:**
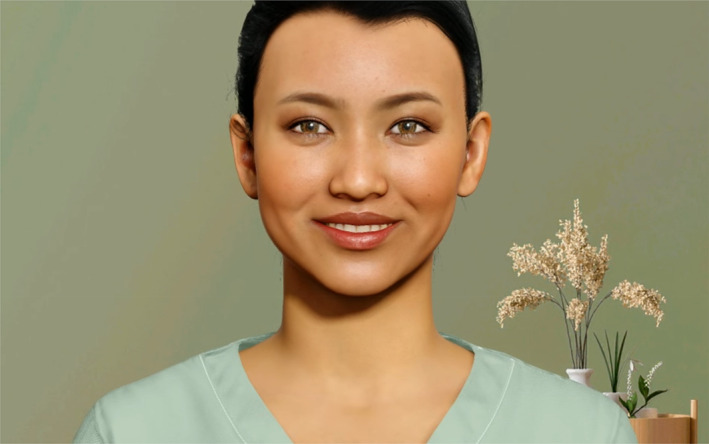
Image of the virtual human GP developed with Soul Machines AI software.

VH non‐verbal communication was AI‐driven and dynamic. The VH used Natural Language Processing (NLP) for speech analysis and facial recognition technology to process user vocal and facial behavior. Based on these inputs, AI generated non‐verbal responses including eye contact, eye movements, hand gestures, facial expressions, and head nodding. AI determined the selection and timing of these behaviors based on its training on human interactions. AI adjusted VH voice tone (pitch, intonation) to match scripted dialog emotional content. The VH GP illustrated in Figure 1 was presented in a neutral position and displayed basic non‐verbal behaviors including nodding, minor head movement, minor arm gestures, and lip movement. When awaiting the participant response, the VH GP maintained eye contact and blinked, simulating active listening. The VH GP offered positive feedback. Verbal communication was scripted; non‐verbal communication was non‐scripted and adaptive. These AI‐driven non‐verbal behaviors, performing a task previously considered requiring human intelligence, constitute an AI feature of this intervention.

This VH configuration differed from other virtual training tools. For example, unlike a pre‐recorded avatar that recited a script, this VH provided real‐time interaction, altering tutorial progression based on user input and testing information retention. In contrast to a text‐based chatbot, this VH integrated an embodied human form with AI‐driven non‐verbal communication, processing facial and vocal cues, engaging in non‐verbal communication, and providing human‐like identity. Generative AI models are non‐scripted, introducing unpredictability, hallucination, higher costs, and regulation challenges in high‐stakes applications; this intervention used a rule‐based, scripted approach for verbal content, prioritizing control, consistency, and task‐specificity. This AI‐driven VH combines scripted verbal content delivered via a rule‐based selection system with AI non‐verbal communication, integrated into a digital human avatar. This design provided a realistic and controlled conversational experience for GP obesity education and communication‐skills training, operating within the constraints of the pre‐programmed script.

### 2.5 Participants and Procedure

A combination of purposive sampling and self‐selection recruitment strategies was employed to obtain a sample of 22 participants. GPs were recruited through direct outreach to a national network and online via platform X. Interested participants contacted the lead author and received a secure Qualtrics link to study materials. Electronic informed consent was obtained, and data were collected through an anonymous online survey. The survey, intervention, and post‐intervention measures were estimated to take approximately 30–45 min to complete. Post‐intervention, six participants withdrew from the study. Consequently, 16 participants completed four of the five post‐intervention measures, and 12 participants provided complete data. This attrition occurred after the intervention but before the completion of all post‐intervention data collection. The attrition rate was noted and considered in the subsequent analysis. Attrition reasons were not collected. Nonetheless, the sample size aligned with recommendations for pilot feasibility trials, which suggested that a sample size of *N* = 12 is suitable when there is no existing data to inform a power calculation [28]. Participants were eligible if they were qualified or in GP training, aged ≥ 18, with experience treating patients with obesity. Participants received no compensation for their participation in this study.

## 3 Materials

### 3.1 Knowledge Acquisition

A 12‐item multiple‐choice questionnaire was developed based on the training materials and piloted; adjustments were made based on planning group feedback. The questionnaire assessed participants' understanding of obesity, weight stigma, and the 5A's framework at two timepoints. Each item had one correct answer, and participants received a score of 1 for each correct response (total scores ranged from 0 to 12). Higher scores indicated greater knowledge acquisition.

### 3.2 Self‐Efficacy Beliefs

Self‐efficacy was assessed using the 12‐item Self‐Efficacy (SE‐12) [29] questionnaire, which measured communication skills regarding weight‐based communication with patients with obesity on a 10‐point Likert‐type scale (0 = very uncertain; 10 = very certain). A total score was obtained for two time points by summing the individual item scores, with higher scores indicating greater self‐efficacy. This scale had a high internal consistency (*α* = 0.89).

### 3.3 Empathy Toward Patients With Obesity and Confidence in Clinical Interactions

Participants' empathy and confidence in clinical interactions were measured using an adapted 14‐item version of the Kushner et al. [30] scale. This scale included three subscales: Negative Stereotyping, Empathy, and Confidence. Items were rated on a five‐point Likert scale (1 = strongly disagree to 5 = strongly agree). Negative Stereotyping scores were reversed, and a total score was obtained by summing across all items, with higher scores reflecting a more positive and confident approach to interacting with patients with obesity. This scale demonstrated good internal consistency (*α* = 0.90).

### 3.4 Virtual Human Perceptions

Perceptions of the VH were measured using the six‐item Agent Persona Instrument‐Revised (API‐R) [31]. This measure assessed participants' views on the VH persona using a five‐point Likert scale, (1 = strongly disagree; 5 = strongly agree). Higher scores indicated more positive perceptions. The API‐R showed strong internal consistency (*α* = 0.85).

### 3.5 Trust in the Virtual Human

Trust in the VH was measured using a modified nine‐item trust scale by Jian et al. [32] This scale used a seven‐point Likert scale (1 = not at all; 7 = extremely), with the first four items negatively worded and reversed for scoring purposes. Total scores were calculated by summing across all items, with higher scores indicating greater trust in the VH. This scale exhibited high internal consistency (*α* = 0.88).

### 3.6 Behavioral Intention

Behavioral intention was measured post‐intervention, using a three‐item scale adapted from Khong et al. [33]. Participants responded using a true/false format to assess their intention to apply the learned 5 As framework and VH tool in practice.

### 3.7 Post‐Intervention Acceptability

Post‐intervention acceptability was evaluated via three researcher‐developed Likert‐scale questions (1 = very negative; 5 = very positive). These questions assessed participants' perceptions regarding: (1) the AI‐driven intervention relative to traditional training modalities; (2) the overall suitability of a VH for medical education; and (3) the participants perceived potential of the virtual GP to directly support patients with weight management in future iterations of the intervention.

## 4 Data Analysis

Descriptive statistics and demographic characteristics were obtained for baseline participant characteristics. Inferential statistical analyses were used to address the research objectives using paired‐sample *t*‐tests to evaluate within‐group mean differences from pre‐to‐post intervention for the primary outcome measures of knowledge, self‐efficacy, and the empathy and confidence in clinical interactions scale score, and two pre‐post subscale comparisons for negative stereotyping and confidence. Average scores from baseline to post‐intervention were compared for each outcome variable, and paired‐sample *t*‐tests were used for normally distributed data. Mean difference (MD) and Cohen's *d* are reported as measures of effect size. To control for multiple comparisons, a Bonferroni correction was applied. With five paired‐sample *t*‐tests conducted, the alpha level was adjusted to 0.01. The feasibility and acceptability of the virtual GP intervention were assessed using descriptive statistics, including means and standard deviations for post‐intervention acceptability questionnaires. All analyses were conducted using SPSS version 27 (IBM Corp., Armonk, NY, USA).

### 4.1 Ethics

Ethical approval for this study was granted by the University of Galway Research Ethics Committee (Ref No: 2023.11.019). Ethical design considerations were integral to the project's conceptualization, particularly regarding the use of AI. Participants were informed about the VH GPs' capabilities and limitations, and an “opt‐out” feature was embedded in the program, allowing participants to withdraw from the study at any point. To mitigate potential biases inherent in AI tools, we employed a value‐sensitive design approach, incorporating planning group feedback on design and delivery. The content was examined in light of the key requirement for trustworthy AI to ensure that the intervention had a universal design that was accessible to all levels of ability and background [34]. This approach aimed to minimize the risk of unintentional biases or discriminatory behavior within the VH programming.

## 5 Results

Following the intervention, six of the 22 recruited general practitioners withdrew. Of the remaining 16 participants, 12 provided complete data, including the knowledge acquisition measure. All available data were used in the analyses to maximize transparency and statistical power. Detailed demographic characteristics are presented below in Table 2.

**TABLE 2:** Participant demographics.

Characteristics and category	Original sample (*N = 22)*	Post‐intervention sample (*n = 16)*	Final post‐intervention sample (*n = 12)*
	*N* (%)	*N* (%)	*N* (%)
Age			
18–25 years	4 (18.2%)	3 (18.7%)	2 (16.7%)
25–34 years	6 (27.3%)	6 (37.5%)	5 (41.7%)
35–44 years	6 (27.3%)	6 (37.5%)	4 (33.3%)
45–54 years	3 (13.6%)		
55+ years	3 (13.6%)	1 (6.3%)	1 (8.3%)
Gender			
Male	6 (27.3%)	5 (31.3%)	4 (33.3%)
Female	16 (72.7%)	11 (68.7%)	8 (66.7%)
Years of experience			
Less than 1 year	4 (18.2%)	3 (18.8%)	2 (16.6%)
1–2 years	7 (31.8%)	7 (43.7%)	5 (41.7%)
2–5 years	11 (50.0%)	6 (37.5%)	5 (41.7%)
Geographic location			
Urban area	15 (68.2%)	9 (56.2%)	6 (50%)
Rural area	7 (31.8%)	7 (43.8%)	6 (50%)

*Note:* Percentages are rounded to the nearest tenth.

### 5.1 Comparison of Means

#### 5.1.1 Knowledge Acquisition

A paired‐sample *t*‐test was conducted on the 12 participants who completed the knowledge acquisition measure. The results indicated a significant increase in knowledge scores from pre‐intervention (*M* = 7.50, SD *=* 2.47) to post‐intervention (*M* = 10.42, SD *=* 1.73), with a mean difference of 2.92 (SE = 0.87), *t* ^*(11)*^ = 3.37*, p* = 0.006. This large effect size (*d* = 0.97) indicated that the VH intervention effectively enhanced knowledge related to obesity, the 5 As framework and structured weight‐based communication.

#### 5.1.2 Self‐Efficacy

Participants who completed both pre‐ and post‐intervention self‐efficacy questionnaires were included in the analysis (*n* = 16). A paired‐samples *t*‐test indicated a significant increase in self‐efficacy scores from pre‐intervention (*M* = 74.88, SD *=* 16.41) to post‐intervention (*M* = 93.25, SD *=* 10.59), *t* ^*(15)*^ = 4.25*, p* = < 0.001, *d* = 1.06. This large effect size indicated that the VH intervention significantly increased participants' perceived confidence in their ability to engage in weight‐based communication.

#### 5.1.3 Empathy and Confidence in Clinical Interactions

Combined empathy and confidence scale scores significantly increased from pre‐intervention (*M* = 41.31, SD *=* 7.00) to post‐intervention (*M* = 48.94, SD *=* 7.76). The VH intervention elicited a mean increase of 7.63, (SE = 2.01), (*t* ^*(15)*^ = 3.79*, p* = 0.002, *d* = 0.95). The scale included a *Negative Stereotyping* subscale which demonstrated a significant decrease in scores from pre‐intervention (*M* = 25.25, SD *=* 6.65) to post‐intervention (*M* = 20.88, SD *=* 6.83), *t* ^*(15)*^ = −3.11*, p* = 0.007, *d* = −0.78, indicating a reduction in negative stereotypes after interacting with the VH. *Confidence* subscale scores significantly increased from pre‐intervention (*M* = 8.75, SD = 2.49) to post‐intervention (*M* = 12.13, SD = 1.5), *t* ^*(15)*^ = 5.04*, p* < 0.001, *d* = 0.67. The VH intervention elicited a mean increase of 3.38 (SE = 1.26) in confidence scores, indicating that the participants' confidence increased after interacting with the VH intervention. Table 3 illustrates these results.

**TABLE 3:** Comparison of pre‐and post‐intervention scores.

Measure	Pre‐intervention (M ± SD)	Post‐intervention (M ± SD)	SE	*t*	*df*	*p*
Knowledge total score	7.50 (2.47)	10.42 (1.73)	0.87	3.37	11	0.006
Self‐efficacy total score	74.88 (16.41)	93.25 (10.59)	4.32	4.25	15	0.001
Empathy total score	41.31 (7.00)	48.94 (7.76)	2.01	3.79	15	0.002
Negative stereotyping subscale	25.25 (6.65)	20.88 (6.83)	1.40	−3.11	15	0.007
Confidence in clinical interactions subscale	8.75 (2.49)	12.13 (1.5)	0.67	5.04	15	0.001

Abbreviations: M = mean; SD = standard deviation; SE = standard error mean.

### 5.2 Post‐Intervention Evaluations

#### 5.2.1 Behavioral Intention

Participants reported intentions to use both the 5 As framework (91.7%) and the VH tool (83.3%) in future practice. A smaller percentage of participants indicated that they would not use the 5 As framework (8.3%) or the VH tool (16.7%).

#### 5.2.2 Acceptability of VH

Participants rated the VH favorably compared to traditional teaching/training methods (*M* = 3.75, SD = 0.71). When asked to compare the VH with traditional teaching/training methods, 37.5% found it equally effective, 50% found it somewhat more effective, and 12.5% found it much more effective. Overall impressions of the VH were positive (*M* = 4.25, SD = 1.04), with 50% reporting “very positive,” 37.5% reporting “somewhat positive,” and 12.5% reporting “negative.” Regarding future use of the VH for patient self‐obesity‐management support, 50% reported “not sure,” 41.7% reported “yes,” and 8.3% were unsure.

#### 5.2.3 Perceptions of the Virtual Human

The API‐R scores indicated positive perceptions of the VH (*M =* 25.50, SD = 2.50). Trust in the VH was moderately high (*M* = 47.38; SD = 7.51), out of a total possible score of 63, suggesting that the VH was perceived as credible and engaging for obesity education and communication skills training.

## 6 Discussion

This pilot feasibility study demonstrated the promising feasibility and preliminary evidence of effectiveness of a VH‐based intervention in improving general practitioners' (GPs) knowledge, self‐efficacy, and empathy toward patients with obesity, as well as their confidence in clinical interactions. These findings underscore the potential of VH technology as an innovative tool for medical education in obesity management.

Participants' evaluations of the VH tool were favorable, particularly when compared to traditional training methods, indicating its potential for engaging general practitioners. Participants also expressed a strong intention to use the VH tool in future practice. This suggested that the tool could be integrated into clinical workflows as an on‐demand, modular training resource, accessible directly from GP clinics or homes. Such an implementation would facilitate flexible, self‐paced learning, allowing for pre‐consultation preparation, post‐consultation reflection, or specific scenario‐based simulations that fit into busy GP schedules. This high acceptability was further evidenced by high API‐R scores and high levels of trust, suggesting that participants found the tool engaging and reliable. The results were supported by findings from a recent study [35], which demonstrated that medical students generally held a favorable attitude toward AI as a learning tool, considering it effective and credible. This cross‐sectional study involving over 700 medical students, highlighted that AI tools were perceived as optimizing study time, providing up‐to‐date medical information, and enhancing the understanding of medical concepts [35]. The observed high levels of trust in this study further supported the potential integration of VH‐based interventions into real‐world healthcare settings [36]. While most participants intended to use the VH tool, some uncertainty was noted, highlighting the need for further research on factors influencing long‐term adoption [37].

The observed increase in knowledge acquisition scores demonstrated the feasibility of the VH training in enhancing GPs' knowledge of obesity and related communication skills. The VH intervention successfully delivered key information on obesity, weight stigma, biopsychosocial factors, and person‐centered communication strategies, demonstrating its ability to convey essential knowledge and skills for patient management [38]. Consistent with our feasibility findings, studies have shown that digital learning tools, including virtual training, effectively improve knowledge and communication skills in medical education [15, 39, 40]. Therefore, the VH tool presented a feasible and effective approach to address existing gaps in obesity management education and communication skills training, ensuring that practitioners were equipped with the knowledge and skills necessary to support their patients.

The significant increase in self‐efficacy scores demonstrated the VH intervention's effectiveness in enhancing GPs' confidence to initiate weight‐related consultations. This finding was critical, as low self‐efficacy was a documented barrier to effective weight management counseling in general practice [9, 41]. Notably, enhanced self‐efficacy was associated with increased frequency and quality of counseling [42]. The specific design of the VH, incorporating interactive and practice‐oriented elements within a controlled virtual environment, effectively facilitated GPs' skill acquisition and bolstered their confidence. Supporting this, studies have consistently shown that virtual and simulation‐based training enhance self‐efficacy among healthcare practitioners across various professions [43, 44]. The findings of this study demonstrated the feasibility of VH technology to provide a practical and scalable approach to address self‐efficacy barriers in general practice.

The VH intervention demonstrated significant improvements in both empathy and confidence during clinical interactions, suggesting a positive influence on GPs' attitudes toward patients with obesity and their ability to conduct sensitive weight‐related consultations. The reduction in negative stereotyping scores was particularly noteworthy given the well‐documented prevalence of weight stigma in healthcare settings, which could compromise patient care [11]. The observed reduction was likely attributable to the VH intervention's comprehensive delivery of content, which included contemporary understandings of obesity [2, 3], the impact of weight stigma on patient‐provider relationships, and a communication skills module designed to support person‐centered weight management [4]. Through education and guided practice within a supportive learning environment, the intervention demonstrated its potential to facilitate empathic communication skills and cultivate more compassionate patient‐provider interactions. This is supported by evidence from studies where participants in virtual training outperformed those in traditional role‐play groups by 5% in measures of empathetic communication skills [45]. Consistent with these findings, Chou et al. [46] reported that nursing students who participated in virtual training communication simulations demonstrated significantly enhanced communication abilities and confidence compared with those receiving traditional video instruction. These results, combined with our current study, indicate that VH interventions can effectively improve not only technical knowledge but also empathy and interpersonal skills, fostering a patient‐centered approach to obesity management. Further research is needed to rigorously evaluate these findings, particularly the observed reduction in weight stigma, to better understand the long‐term impact and generalizability of VH interventions in this context.

## 7 Limitations and Future Directions

Despite promising feasibility results, this pilot study had inherent limitations. The sample size, while adequate for a pilot study, was smaller than anticipated due to participant attrition. Attrition is a common challenge in studies involving this cohort [6, 47]. Although specific reasons for attrition were not collected, the study's duration (∼45 min) may have contributed to participant withdrawal. Nonetheless, the data obtained will inform power calculations for future efficacy trials. The reliance on self‐report measures introduced the potential for social desirability bias. Furthermore, the absence of a control group limited our ability to definitively assess the VH intervention's effectiveness relative to other training methods. This highlighted the need for future efficacy trials with appropriate control conditions to establish the intervention's comparative effectiveness. The short duration of the study restricted our ability to evaluate long‐term impacts on the outcome measures. Future research should prioritize larger efficacy trials with control conditions and incorporate qualitative insights to explore participant experiences in greater depth.

The scripted nature of the VH's verbal responses was another consideration in this study. The scripted approach ensured consistency and controlled content delivery. However, it limited dynamic conversational adaptability beyond predefined pathways. Nonetheless, this design choice facilitated the evaluation of empirically derived content while specifically avoiding the unpredictability and potential for hallucination inherent in current generative AI models; thus, the study prioritized content standardization, safety, and trustworthiness over novelty. Future models could integrate more sophisticated rule‐based systems or conditionally generative elements within strict clinical and ethical guidelines. This would allow for dynamic interaction, balancing it with the need for content fidelity and reliability in the standardization of medical education and training.

## 8 Conclusion

The findings of this pilot feasibility study demonstrated that the VH tool presented a unique and promising solution for delivering obesity education and enhancing the understanding of weight‐related communication skills among general practitioners. In offering an interactive and safe learning environment, the VH enabled GPs to acquire knowledge of a person‐centered communication framework, with immediate feedback through quizzes. This approach holds significant potential to complement traditional training methods, particularly in time‐constrained general practice settings. These results suggest that VH technology could be effectively integrated into continuous professional development programs, offering a scalable and accessible means to improve the quality of weight‐related communication and patient outcomes. Future research should focus on evaluating the long‐term impact of VH interventions in real‐world clinical settings and explore the optimal integration of this technology into existing training curricula.

## Conflicts of Interest

L.R. is an ASOI committee member. M.C. reports honoraria for educational events or conference attendance from Novo Nordisk and Consilient Health and was a member of a Novo Nordisk advisory board. He is a member of the Irish ONCP Clinical Advisory Group and ASOI. S.B. reports funding to ICPO from the HSE, Novo Nordisk, and the European Coalition for People Living with Obesity (ECPO) and consulting fees or honoraria from Diabetes Ireland, ECPO, and Novo Nordisk. S.B. is the Executive Director of ICPO and the Secretary of ECPO. The remaining authors have no conflict of interest to declare.

## Data Availability

The authors confirm that the data supporting the findings of this study are available within the article. Raw datasets are available from the corresponding author upon reasonable request. The study materials (i.e., measures, scripts) are available on the Open Science Framework (OSF) repository, https://osf.io/c24sm/files/osfstorage.
